# Left Anterior Fascicular Block After Transcatheter Closure of Ventricular Septal Defect in Children

**DOI:** 10.3389/fcvm.2021.609531

**Published:** 2021-06-11

**Authors:** Zhijun Wu, Penghui Yang, Ping Xiang, Xiaojuan Ji, Jie Tian, Mi Li

**Affiliations:** ^1^Ministry of Education Key Laboratory of Child Development and Disorders, Chongqing, China; ^2^China International Science and Technology Cooperation Base of Child Development and Critical Disorders, Chongqing, China; ^3^Department of Cardiovascular Medicine, Children's Hospital of Chongqing Medical University, Chongqing, China; ^4^National Clinical Research Center for Child Health and Disorders, Chongqing, China; ^5^Chongqing Key Laboratory of Pediatrics, Chongqing, China; ^6^Department of Ultrasound, Children's Hospital of Chongqing Medical University, Chongqing, China

**Keywords:** ventricular septal defect, transcatheter closure, left anterior fascicular block, arrhythmias, children

## Abstract

**Background:** Arrhythmia is the most common complication after transcatheter closure of a ventricular septal defect (VSD). However, the effects of postprocedural left anterior fascicular block are not clear. This study presents the clinical characteristics, prognosis, and related risk factors of left anterior fascicular block after transcatheter closure of a VSD in children.

**Methods:** The clinical and follow-up data of the patients in the Heart Center of Children's Hospital of Chongqing Medical University from June 2009 to October 2018 were reviewed. And 30 cases were eligible out of all 1,371 cases.

**Results:** An electrocardiogram showed a left anterior fascicular block within 3 days, and most patients gradually returned to normal within 1–2 years, showing a dynamic change. Left ventricular end-diastolic dimension *Z*-score ranged from −2 to 2 in all children, and no decrease of left ventricular ejection fraction was found in all children. The high ratio between VSD size and body surface area [*p* < 0.05, odds ratio (OR) 2.6, 95% CI: 1.136–6.113] and large diameter difference between the occluder size and VSD size (*p* < 0.05, OR 2.1, 95% CI: 1.036–4.609) were independent risk factors for postprocedural left anterior fascicular block.

**Conclusions:** The incidence of postprocedural left anterior fascicular block is not that low, and the overall prognosis is quite good at the current follow-up stage. No progressive severity has been found, such as complete left bundle branch block, double (triple) bundle branch block, and atrioventricular block, to have an influence on cardiac systolic and diastolic function.

## Introduction

Ventricular septal defect (VSD) is one of the most common congenital heart diseases (CHDs) in children. Transcatheter closure of a VSD has been an alternative to surgery because of its advantages such as less trauma, quicker recovery, and shorter hospitalization days. However, what we cannot ignore is postprocedural arrhythmias, the most common complication after the closure, including atrioventricular block (AVB), bundle branch block (BBB), non-sinus tachycardia, and frequent premature contractions, etc. (1, 2). Given the importance of the left ventricle to the whole circulation system, many other postprocedural blocks, such as the second degree type II AVB or above and complete left bundle branch block (CLBBB) that have an influence on hemodynamics and left ventricular function to varying degrees, were specialized in and described in detail previously (3–5). Left anterior fascicular block (LAFB), or left anterior bundle branch block, one type of left bundle branch block (LBBB), can result in a certain degree of ventricular asynchrony theoretically affecting hemodynamics and left ventricular function; more attention should be paid to this aspect as well. However, reports describing the clinical characteristics, prognosis, and related risk factors of postprocedural LAFB are lacking, bringing difficulties in clinical management. Hence, we aim to determine the clinical characteristics, follow-up outcomes, and related risk factors of LAFB after transcatheter closure of a VSD in children.

## Patients and Methods

### Study Population

From June 2009 to October 2018, a total of 1,371 VSD patients underwent transcatheter closure successfully at the Heart Center of Children's Hospital of Chongqing Medical University. A total of 255 cases of arrhythmias occurred after closure, and there were 129 cases with BBB including 30 cases with LAFB.

Inclusion criteria were as follows: (1) Age <18 years old, (2) the preprocedural diagnosis of VSD had been proven by echocardiogram, (3) patients who successfully underwent transcatheter closure, and (4) new LAFB change in electrocardiogram (ECG) after closure. Exclusion criteria were patients who failed to undergo transcatheter closure and had preprocedural LAFB.

### Study Design

The study was approved by the Ethics Committee of the Children's Hospital of Chongqing Medical University (No. 2020-10). All cases followed domestic guidelines of transcatheter intervention therapy for CHD (6, 7). Before the procedure, informed consent was obtained from guardians of the patients, and all patients underwent ECG, echocardiogram, and other related examinations. Left ventricular angiography was performed to determine the anatomical type and size of the VSD and relationship between VSD and aortic rim on procedure, and an appropriate occluder was selected for the defect. All operators were experienced, having worked on CHD transcatheters for more than 10 years, and the methods for transcatheter closure of VSD were all the same and followed the domestic guidelines. A routine ECG monitoring was performed within 24 h after the procedure, and ECG was re-performed daily until discharge. The echocardiogram was re-performed the next day after the closure. In months 1, 3, and 6 and every year of follow-up, we recorded left ventricular ejection fraction (LVEF), left ventricular end-diastolic dimension (LVEDD) outcomes from the echocardiogram, and ECG. Several patients who failed to return to the hospital on time would collect their follow-up data by fax or mail.

### Statistical Analysis

All tests were performed by the IBM SPSS Statistics Version 22 statistical software package (SPSS Inc., Chicago, Illinois, USA). Categorical variables were expressed as count and percentage, and continuous variables were expressed as means ± standard deviations with ranges. The difference was tested with χ^2^-test, and logistic regression analysis was performed to identify independent risk factors of postprocedural LAFB. The β coefficient, odds ratio (OR), and the corresponding 95% CI were calculated at the same time. *p*-value below 0.05 was considered significant.

## Results

### General Information

Among the 30 cases, there were 17 males and 13 females with an average age of 42 ± 25 months (range 23–105 months), average weight 14.4 ± 3.7 kg, average body surface area (BSA): 0.60 ± 0.13 m^2^. All the patients had LAFB within 3 days of closure, including 25 cases on the next day, three cases on the second day, and two cases on the third day. The general information of the patients is shown in Table 1.

**Table 1:** General information.

**Variables**	
Gender [male/female, *n* (%)]	17/13 (56.7/43.3)
Age (month)	55 ± 25
Weight (kg)	14.4 ± 3.7
BSA (m^2^)	0.60 ± 0.13
VSD type [*n* %]	
pmVSD	24 (80.0)
pmVSD with pseudo aneurysm	5 (16.7)
mVSD	1 (3.3)
VSD size (x ± s, mm) (Angiography)	3.7 ± 1.8
d_VSD_/BSA (mm/m^2^)	5.6 ± 2.1
Occluder type [*n* %]	
Symmetric occluder	25 (83.3)
Eccentric occlude	2 (6.7)
Small-waist occlude	2 (6.7)
Muscular occluder	1 (3.3)
Occluder size (mm)	6.9 ± 1.8
DDOV (mm)	3.3 ± 1.0
Operation time (min)	45.9 ± 15.3

*BSA, body surface area; pmVSD, perimembranous VSD; mVSD, muscular VSD; d_VSD_/BSA, the ratio between VSD size and body surface area; DDOV, diameter difference between the occluder size and VSD size*.

### Follow-Up

With a follow-up duration of 1–112 months and a median time of 30 months, 23 cases were followed up for more than 1 year. The follow-up rate of 1, 6 months, and 1 year after the closure were 93.3% (28/30), 83.3% (25/30), and 76.7% (23/30), respectively. The postprocedural ECG showed LAFB and complete right bundle branch block (CRBBB) in three cases. During the follow-up duration, one of the ECG procedures showed left axis deviation from 1 month to 2 years after the closure and returned to normal after 3 years. One of the ECG procedures showed LAFB and incomplete right bundle branch block (IRBBB) after 1 month and returned to normal after 3 months. One of the ECGs showed CRBBB from 1 month to 1 year and returned to normal after 2 years.

During the follow-up, the LVEDD of all the patients fluctuated from 29 to 42 mm, LVEDD *Z*-score ranged from 0.6 to 1.9. LVEDD *Z*-score was calculated according to the Boston Children's Hospital, and the normal *Z*-score was between −2 and 2, and the purpose of the *Z*-score was to correct the effect of BSA and weight on LVEDD (8, 9). The LVEF fluctuated from 59 to 71% in all patients. The follow-up data of ECG, LVEDD *Z*-score, and LVEF are shown in Tables 2, 3.

**Table 2:** Follow-up data of ECG [*n* (%)].

	**1–3 d** **(*n* = 30)**	**1 m** **(*n* = 28)**	**3 m** **(*n* = 28)**	**6 m** **(*n* = 25)**	**1 y** **(*n* = 23)**	**2 y** **(*n* = 19)**	**3 y** **(*n* = 15)**
LAFB	27 (90.0)	13 (46.4)	9 (32.1)	5 (20.0)	3 (13.1)	1 (5.3)	1 (6.7)
LAFB and CRBBB	3 (10.0)	0	0	0	0	0	0
LAFB and IRBBB	0	1 (3.6)	0	0	0	0	0
CRBBB	0	1 (3.6)	1 (3.6)	1 (4.0)	1 (4.3)	0	0
Left axis deviation	0	7 (25.0)	7 (25.0)	6 (24.0)	4 (17.4)	1 (5.3)	0
Normal	0	6 (21.4)	11 (39.3)	13 (52.0)	15 (65.2)	17 (89.4)	14 (93.3)

*d, day(s); m, month(s); y, year(s); LAFB, left anterior fascicular block; CRBBB, complete right bundle branch block; IRBBB, incomplete right bundle branch block*.

**Table 3:** Follow-up data of LVEDD *Z*-score and LVEF (%).

	**1–3 d** **(*n* = 30)**	**1 m** **(*n* = 28)**	**3 m** **(*n* = 28)**	**6 m** **(*n* = 25)**	**1 y** **(*n* = 23)**	**2 y** **(*n* = 19)**	**3 y** **(*n* = 15)**
LVEDD *Z*-score	1.6 ± 0.3	1.2 ± 0.2	1.1 ± 0.3	1.2 ± 0.3	1.0 ± 0.1	0.9 ± 0.3	0.9 ± 0.2
LVEF (%)	63.4 ± 3.6	64.1 ± 3.2	64.9 ± 3.9	64.6 ± 3.1	65.1 ± 2.9	64.4 ± 3.1	64.8 ± 2.6

*d, day(s); m, month(s); y, year(s); LVEDD, left ventricular end-diastolic dimension; LVEF, left ventricular ejection fraction*.

### Risk Factors for Postprocedural LAFB

Logistic regression analysis was conducted to determine the risk factors for postprocedural LAFB. LAFB was used as the dependent variable. Significant variables in univariate analysis including age, BSA, the ratio between VSD size and BSA (d_VSD_/BSA), occluder size, diameter difference between the occluder size and VSD size (DDOV), and operation time were introduced into the logistic model. Binary logistic regression analysis revealed that the high ratio between VSD size and BSA (d_VSD_/BSA) (*p* < 0.05, OR 2.6, 95% CI: 1.136–6.113) and a large DDOV (*p* < 0.05, OR 2.1, 95% CI: 1.036–4.609) were independent risk factors for postprocedural LAFB. The details are shown in Table 4.

**Table 4:** Logistic regression analysis of risk factors for LAFB after transcatheter closure of VSD in children.

	**Non-arrhythmia Group (*n* = 1,116)**	**LAFB group (*n* = 30)**	**β**	***p*-value**	**95% CI**
Age	48 ± 19	55 ± 25	−0.058	0.154	0.871–1.022
BSA	0.66 ± 0.17	0.60 ± 0.13	1.953	0.180	2.802–3.305
VSD size	3.3 ± 1.1	3.7 ± 1.8	−1.226	0.085	0.073–1.183
d_VSD_/BSA	5.2 ± 2.3	5.6 ± 2.1	0.969	0.018	1.136–6.113
Occluder size	6.2 ± 1.0	6.9 ± 1.8	−0.165	0.670	0.397–1.812
DDOV	3.0 ± 1.1	3.3 ± 1.0	0.363	0.024	1.036–4.609
Operation time	46.4± 18.6	45.9 ± 15.3	−0.032	0.236	0.918–1.021

*β, logistic correlation coefficient; 95% CI, 95% confidence interval*.

## Discussion

In the absence of structural heart disease, LAFB is thought of as a benign ECG finding, though it can cause a certain degree of ventricular asynchrony. LAFB is common in coronary heart disease and hypertension in adults (10–12). Mandyam et al. (10) reported that LAFB had an increased risk of atrial fibrillation, heart failure, and all-cause and cardiovascular mortality in individuals without overt cardiovascular diseases. Paradoxically, Nielsen et al. (11) put forward a different conclusion that LAFB can only increase the risk of all-cause mortality. But both reports found no progressive severity of LAFB to CLBBB, double (triple) bundle branch block, and AVB.

However, what about children with LAFB with a structural heart disease, such as VSD? It needs more attention. LAFB is always associated with ventricular septum problems in children. On one hand, during spontaneous closure of VSD, it may affect the cardiac conduction system, especially the left bundle branch and its divisions. On the other hand, from the perspective of anatomy, the left anterior bundle branch is a slender branch of the left bundle branch, which runs superficially in the ventricular septum, and thus it is more prone to injury than the left posterior bundle branch when undergoing transcatheter closure of the VSD. Therefore, we can foresee that either a trail building operation or occluder compression may lead to edema of the surrounding tissue or direct injury.

**Figure 1:**
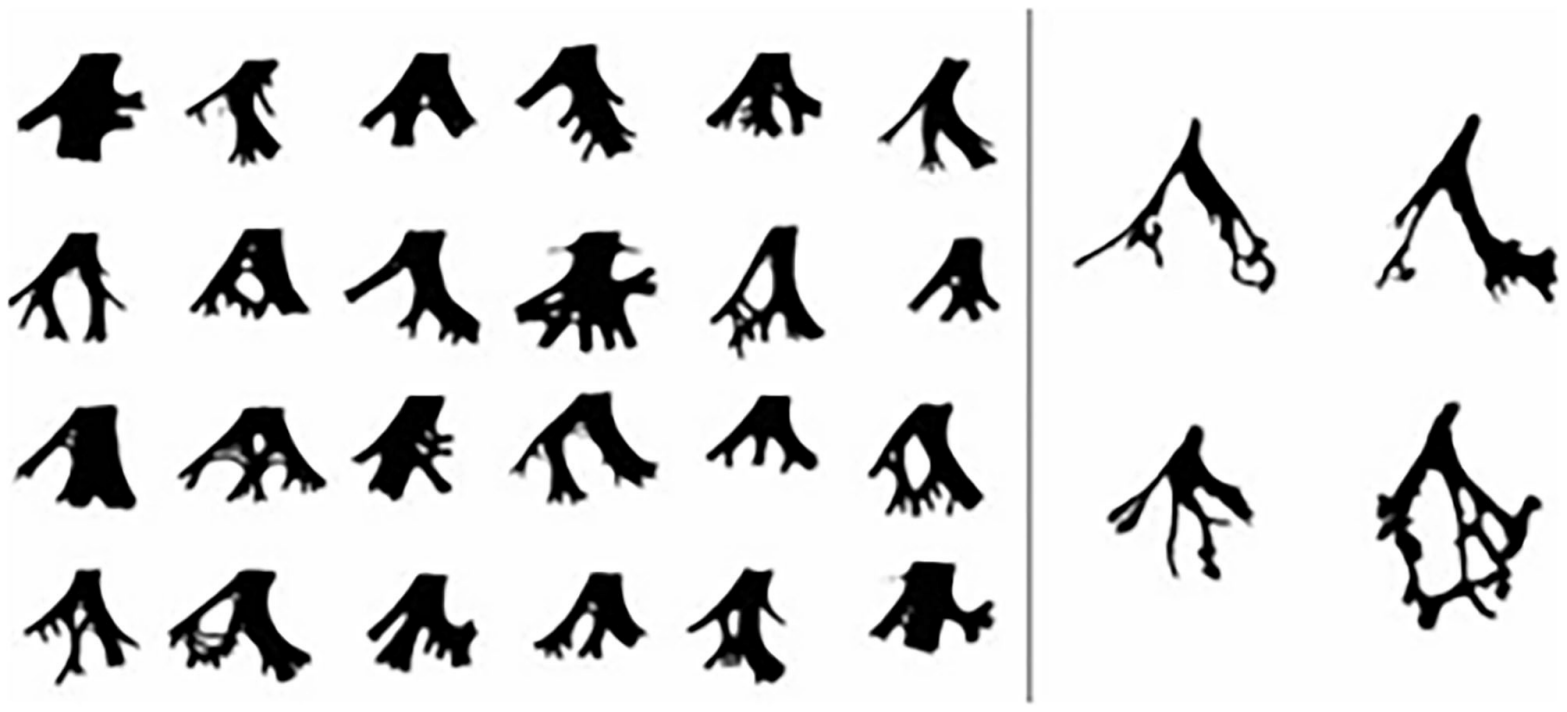
The anatomy type of LBB and its divisions Left, Diagrammatic sketches of the LBB. Right, 4 prototypes of LBB.

The incidence of postprocedural LAFB ranges from 3.6 to 5.5% as reported by other centers (13–15). Our results indicate that postprocedural LAFB has an incidence of 2.2% (30/1,371) among all patients and 11.8% (30/255) among all postprocedural arrhythmias, and it is not that low, especially among postprocedural BBB (23.3%, 30/129). The difference of incidence of postprocedural LAFB between our center and other centers may be caused by two reasons. On one hand, this is a retrospective study conducted at the local center; the data cannot be the same across all centers. On the other hand, the patients in this study were the rest part that knocked off the 13 cases one who existed preprocedural LAFB. There were 1,061 cases of perimembranous ventricular septal defect (pmVSD) and 1,033 cases of selected symmetric occluder, accounting for 77.4% and 75.3% of the total number of surgery, respectively. Similarly, most postprocedural LAFB came from pmVSD and selected symmetrical occlude patients. Thus, VSD type and occluder type are not specific for postprocedural LAFB compared with the patients who underwent transcatheter closure of a VSD at the same time. But one case in the study attracted our attention: a muscular ventricular septal defect (mVSD) also had a postprocedural LAFB. We recognize that mVSD does not easily cause BBB because of its anatomical relationship between cardiac conduction systems. Arora et al. (16) expressed the same opinion in his study on mVSD during follow-up. So, why did this patient suffer postprocedural LAFB? We evaluated and analyzed her general information, including age, BSA, ratio between VSD size and BSA (d_VSD_/BSA), occluder size, DDOV, and operation time. Unfortunately, we found nothing special. As we know, it is difficult to build up an association between electrocardiographic and pathological conduction disturbances, and left bundle branch and its divisions vary one to another (Figure 1) (17). So, we found that ECG presented as a fixed pattern, such as LAFB after transcatheter closure of mVSD in the study, which we cannot explain from normal anatomical and pathological perspectives. Is it possible that this patient has a rare anatomy type or anatomical variation of LBB and its divisions after excluding operative injury.

LV is important to the whole circulation system, and thus we paid attention to arrhythmias that have negative effects on LV function, such as AVB and CLBBB. But both CLBBB and LAFB are the one of LBBB, and with the development of the theory of ventricular systolic synchronization, it is generally believed that CLBBB can cause left ventricular contraction disorder, left and right ventricular non-synchronous contraction, left ventricular and ventricular septum contradictory movement, and eventually lead to heart failure and death (9). Similarly, does LAFB have these problems too? We can see from the follow-up data that in most cases, including the patients with postprocedural double bundle branch block (LAFB and CRBBB), ECG would gradually return to normal with a dynamic change of *LAFB—left axis deviation—normal*. And no LAFB patients displayed progressive severity to CLBBB, double (triple) bundle branch block, and AVB until the follow-up deadline. This is what Mandyam et al. (10) and Nielsen et al. (11) concluded too. But LAFB lasts in a few individuals and needs long-term follow-up. We can see that most LAFBs are a transient ECG change like other postprocedural arrhythmias. The reason is that edema of the myocardium or injury caused by an operation or occluder compression achieves recovery gradually as time goes by, and then the conduction system recovers too. At current follow-up, no patient LVEDD *Z*-score was above 2, and there was no decrease of LVEF in any patients. This may suggest that postprocedural LAFB is a benign ECG finding even with structural heart disease. The reason is related to the domination district of the left anterior bundle branch, which mainly activates the upper left ventricle, causing the left ventricle to contract asynchronously in a small area, and it is not enough to have an effect on LV function and hemodynamics.

Age, body mass index (BMI), VSD size and type, occluder size and type, and operation time were associated with postprocedural arrhythmias in other studies, but there were no recognized risk factors to predict postprocedural outcomes and prognosis; the outcomes were even controversial (2, 13, 18). Our study indicated that the high ratio between VSD size and BSA (d_VSD_/BSA) (*p* < 0.05, OR 2.6, 95% CI: 1.136–6.113) was an independent risk factor for postprocedural LAFB. In other words, after correcting VSD size based on BSA in the LAFB group and non-arrhythmia group, a relatively or absolutely larger VSD size is more likely to affect the conduction function of the left anterior bundle branch. With this is known before transcatheter closure of a VSD, it may be beneficial to suggest that the patient turn to surgical closure. More research is needed on how high the ratio is. A large DDOV (*p* < 0.05, OR 2.1, 95% CI: 1.036–4.609) was also an independent risk factor for postprocedural LAFB. The larger the difference, the greater the choice of occluder size, which leads to more serious compression of the ventricular septum and conduction system around the defect, resulting in direct or indirect damage to the left anterior bundle branch.

## Conclusion

The postprocedural LAFB is not a rare arrhythmia complication after transcatheter closure of a VSD in children. And luckily, no progressive severity of LAFB has been found such as CLBBB, double (triple) bundle branch block, and AVB so as to have an influence on cardiac systolic and diastolic function.

## Data Availability Statement

The original contributions presented in the study are included in the article/supplementary material, further inquiries can be directed to the corresponding author.

## Ethics Statement

The study was approved by the Ethics Committee of Children's Hospital of Chongqing Medical University (No. 2020-10). Written informed consent to participate in this study was provided by the participants' legal guardian/next of kin. Written informed consent was obtained from the individual(s), and minor(s)' legal guardian/next of kin, for the publication of any potentially identifiable images or data included in this article.

## Author Contributions

ZW and PY analyzed and interpreted the patient data of the cases. PX, XJ, JT, and ML contributed to the intellectual content of this manuscript. ZW, PY, and ML were major contributors in writing the manuscript. All authors contributed to the article and approved the submitted version.

## Conflict of Interest

The authors declare that the research was conducted in the absence of any commercial or financial relationships that could be construed as a potential conflict of interest.
